# Pancreatic lymphangioma in an adolescent male managed without surgery: a rare case report

**DOI:** 10.1093/jscr/rjaf629

**Published:** 2025-08-21

**Authors:** Deedat Safeer, Nilesh Fernandopulle, Chathuranga Keppetiyagama, Duminda Subasinghe

**Affiliations:** Department of Surgery, Faculty of Medicine, University of Colombo, No. 25, P.O. Box 271, Kynsey Road, Colombo 08, Sri Lanka; Department of Surgery, Faculty of Medicine, University of Colombo, No. 25, P.O. Box 271, Kynsey Road, Colombo 08, Sri Lanka; University Surgical Unit, The National Hospital of Sri Lanka, Colombo 10, Sri Lanka; Gastroenterological Surgical Unit, The National Hospital - Kandy, Kandy 20000, Sri Lanka; Department of Surgery, Faculty of Medicine, University of Colombo, No. 25, P.O. Box 271, Kynsey Road, Colombo 08, Sri Lanka; University Surgical Unit, The National Hospital of Sri Lanka, Colombo 10, Sri Lanka

**Keywords:** pancreatic lymphangioma, case report, conservative management

## Abstract

Pancreatic lymphangiomas are extremely rare benign cystic lesions of the pancreas, with fewer than 100 cases reported in the literature. They can mimic other cystic pancreatic neoplasms, posing a diagnostic challenge. This case adds to the limited literature and supports conservative management as a safe option in selected patients. A 15-year-old South Asian male presented with right-sided upper abdominal pain persisting for 5 months. Imaging revealed a large, multiloculated cystic lesion involving the neck and body of the pancreas. Aspiration cytology and cyst fluid analysis (low carcinoembryonic antigen, elevated amylase, lymphocytes) were suggestive of pancreatic lymphangioma. The patient became asymptomatic and was managed conservatively with close monitoring. No surgical intervention was performed. This case demonstrates that conservative management of pancreatic lymphangiomas may be appropriate in asymptomatic or minimally symptomatic adolescents with benign imaging and biochemical features. It reinforces the importance of individualized treatment to avoid unnecessary surgery.

## Introduction

Lymphangiomas are rare benign malformations of the lymphatic system. Pancreatic lymphangiomas are particularly uncommon, with fewer than 100 cases reported in the literature. These lesions can mimic other cystic pancreatic neoplasms, making accurate diagnosis essential to exclude malignancy. Traditionally, surgical resection has been the treatment of choice, especially in symptomatic patients or when adjacent structures are compromised. However, conservative management is increasingly recognized as a viable option in asymptomatic or minimally symptomatic cases, as these lesions may regress spontaneously, thereby avoiding unnecessary surgical intervention [1].

## Case presentation

A 15-year-old South Asian male presented with right-sided upper abdominal pain persisting for 5 months, without associated symptoms such as fever, vomiting or weight loss. Abdominal examination was unremarkable. The patient was previously well and had no relevant family history. Laboratory investigations, including serum amylase, were within normal limits.

**Figure 1:**
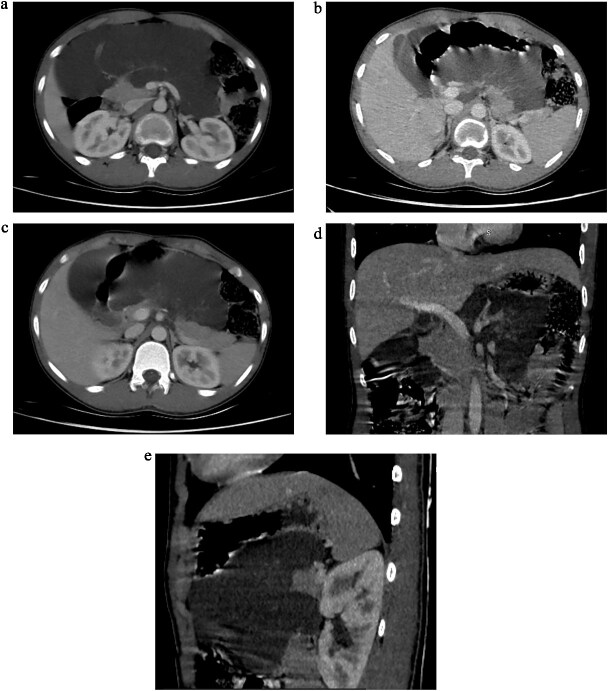
(a) Axial sections of CT abdomen (arterial phase) showing cystic mass in relation to pancreas. (b) Axial sections of CT abdomen (arterial phase) showing cystic mass in relation to pancreas. (c) Axial sections of CT abdomen (venous phase) showing cystic mass in relation to pancreas. (d) Coronal section of CT abdomen (venous phase) showing cystic mass in relation to body and tail of the pancreas. (e) Sagittal section of CT abdomen (venous phase) showing cystic mass in relation to body and tail of the pancreas.

Initial ultrasonography (USS) revealed multiple cystic lesions with parenchymal distortion in the tail of the pancreas. A contrast-enhanced computed tomography (CT) scan showed a large, lobulated, nonenhancing hypodense lesion (6.6 × 16 × 16 cm) with internal septations involving the neck and body of the pancreas. The lesion extended superiorly to the liver hilum and greater curvature of the stomach, anterolaterally to the anterior abdominal wall, and inferiorly to the umbilicus level (L4 vertebra), with no evidence of invasion (Fig. 1a–e). Differential diagnoses included cystic neoplasm and lymphangioma.

Aspiration cytology revealed dark yellow, slightly turbid, alkaline fluid with a protein concentration of 5.91 g/l and glucose of 45 mg/dl. Cytological analysis revealed a mixed inflammatory cell population, comprising numerous macrophages—some containing hemosiderin granules—alongside lymphocytes and neutrophils. No cellular atypia was observed, and the smear was interpreted as benign inflammatory in nature.

A follow-up ultrasound scan revealed a multiloculated cystic lesion with thin septations involving the head, body, and tail of the pancreas. The largest component, measuring 7.4 × 6.6 cm, was located in relation to the head of the pancreas. An interventional radiology review was sought to consider reaspiration; however, it was deemed unhelpful. Surgery with sclerotherapy was recommended as the preferred approach. As digital subtraction angiography (DSA) was not available at the institution, the patient was referred to our center for further management.

**Figure 2:**
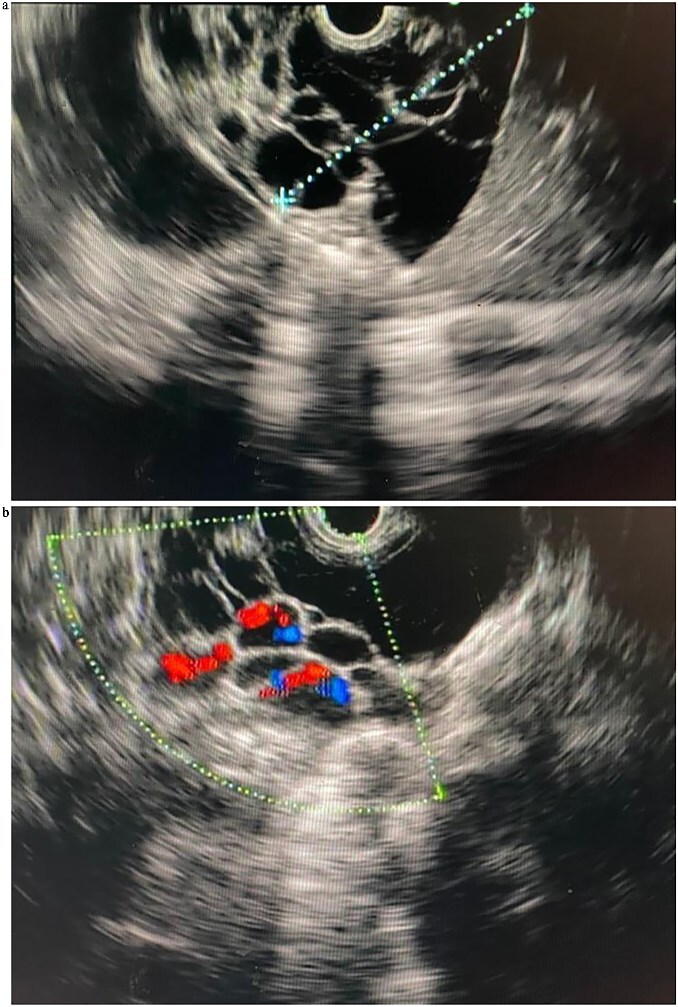
(a) Linear EUS (Olympus ME 1) views of the pancreatic lymphangioma, seen as cystic lesions in the pancreatic parenchyma. (b) Linear EUS (Doppler) (Olympus ME 1) views of the pancreatic lymphangioma, seen as cystic lesions in the pancreatic parenchyma.

Endoscopic ultrasound (EUS) at our center revealed a large, multiseptated cystic lesion posterior to a structurally normal pancreas (Fig. 2a and b). Aspiration yielded 6 ml of yellowish serous fluid. Biochemical analysis showed low carcinoembryonic antigen (CEA 2.39 ng/ml) and elevated amylase (443 U/l); cytology revealed scattered cyst macrophages and lymphocytes only consistent with pancreatic lymphangioma.

Although initially symptomatic, the patient became asymptomatic during the course of conservative management. Follow-up ultrasound imaging demonstrated a slight reduction in the size of the lesion. In the absence of new symptoms or complications, a decision was made to continue with clinic-based observation. The patient remains asymptomatic at 6 months following EUS-guided aspiration.

## Discussion

Pancreatic lymphangiomas are the rarest type of lymphangioma accounting for <1% of all pancreatic neoplasms and are often discovered incidentally or during evaluation of nonspecific abdominal symptoms [1]. Imaging modalities such as CT and magnetic resonance imaging (MRI) are essential in assessing lesion characteristics, but definitive diagnosis often requires aspiration and fluid analysis.

Patients presenting with persistent, nonspecific abdominal pain unresponsive to first-line treatment should undergo imaging as part of their evaluation. The initial modality is typically abdominal USS, which may reveal a multiloculated, anechoic, or hypoechoic cystic lesion with thin septations. While USS is useful as a first-line investigation, it may not adequately characterize the lesion or delineate its relationship to adjacent structures, particularly in retroperitoneal organs like the pancreas. However, in our case, due to the large size of the lesion, it was readily visualized on abdominal ultrasound.

Following the detection of a pancreatic cyst or in cases with high clinical suspicion, cross-sectional imaging is recommended. This is most commonly achieved using contrast-enhanced MRI or magnetic resonance cholangiopancreatography (MRCP), which provide detailed anatomical and ductal assessment. For patients unable to undergo MRI/MRCP, a dedicated pancreatic protocol CT scan serves as a suitable alternative. These imaging modalities help determine the cyst type and assess for high-risk features of malignancy—such as cysts > 3 cm, presence of a solid component, or main pancreatic duct dilation [2].

In our case, due to logistic limitations, a contrast-enhanced CT (CECT) was performed. It revealed a large, lobulated, nonenhancing lesion involving the neck and body of the pancreas. Typical CECT findings of pancreatic lymphangioma, as described in the literature, include a multilocular, multiseptated, and nonenhancing cystic mass [1, 3].

EUS was subsequently performed and proved valuable for further characterization. EUS identified a multiloculated, avascular cystic lesion with thin septations—features that are highly suggestive, though not pathognomonic, of pancreatic lymphangioma. A key advantage of EUS lies in its ability to facilitate fine-needle aspiration (FNA) for biochemical and cytological analysis. Fluid from pancreatic lymphangiomas is typically straw colored and known to demonstrate normal CEA levels, high amylase levels, and elevated levels of lactate dehydrogenase and triglycerides [1, 4]. Our sample showed normal CEA levels and high amylase levels and the aspirated fluid was dark yellow and slightly turbid. Cytological analysis revealed a white blood cell population in a benign inflammatory smear, with no atypia. These findings, in combination with the clinical and radiological context, supported the diagnosis of pancreatic lymphangioma.

An important differential diagnosis, particularly in male patients, is the pancreatic lymphoepithelial cyst (LEC), which can have overlapping imaging appearances with lymphangiomas on CECT [5]. LECs are benign, keratin-filled cysts lined by squamous epithelium and surrounded by lymphoid tissue. On CECT, LECs show enhancement of the wall and septum of the cysts, whereas the cysts themselves show uniform low density without enhancement [6] and contain thick keratinous or proteinaceous material rather than chylous fluid. Aspiration from LECs typically reveals high cholesterol content, low triglycerides, and acellular squamous debris [7], in contrast to the lymphocyte-rich, triglyceride-dominant fluid of lymphangiomas.

In our patient, a diagnosis of pancreatic lymphangioma was made based on a composite assessment of clinical presentation, imaging findings from ultrasound, CECT, EUS and cyst fluid analysis.

Surgical management typically includes cyst excision, distal pancreatectomy, or more extensive procedures such as pancreatoduodenectomy, depending on the lesion’s size and location. Complete resection is usually curative, while partial excision or simple aspiration carries a risk of recurrence [1].

However, in cases where the lesion exhibits benign features, is noninvasive, and the patient remains asymptomatic, conservative management may be a safe and effective alternative [8]. In pediatric patients, this approach also spares pancreatic function and reduces the morbidity associated with major surgery.

This case supports a nonsurgical approach in select patients with pancreatic lymphangiomas. The patient’s clinical improvement, stable imaging findings, and absence of complications provide further evidence that individualized, conservative strategies can be both safe and effective.

## Conclusion

Although rare, pancreatic lymphangiomas should be considered in the differential diagnosis of cystic pancreatic lesions in adolescents. Conservative management with close monitoring may be appropriate in asymptomatic or improving cases, avoiding unnecessary surgical risks. This case adds to the growing evidence supporting individualized, nonoperative strategies in managing pancreatic lymphangiomas.

## Data Availability

Not applicable.
